# Polyoxidonium^®^ Activates Cytotoxic Lymphocyte Responses Through Dendritic Cell Maturation: Clinical Effects in Breast Cancer

**DOI:** 10.3389/fimmu.2019.02693

**Published:** 2019-11-28

**Authors:** Catherine Alexia, Mailys Cren, Pascale Louis-Plence, Dang-Nghiem Vo, Yasamine El Ahmadi, Emilie Dufourcq-Lopez, Zhao-Yang Lu, Javier Hernandez, Farkhad Shamilov, Olga Chernysheva, M. Vasilieva, I. Vorotnikov, Yana Vishnevskay, Nikolay Tupitsyn, Jean-François Rossi, Martin Villalba

**Affiliations:** ^1^IRMB, University of Montpellier, INSERM, Montpellier, France; ^2^CHU Montpellier, Montpellier, France; ^3^Federal State Budgetary Institute “N.N. Blokhin National Oncology Research Center” of the Ministry of Health of Russian Federation, Moscow, Russia; ^4^Voronezh Oncology Dispansery, Vronezh, Russia; ^5^Institut Sainte Catherine, Avignon, France; ^6^Université de Montpellier I, UFR Médecine, Montpellier, France; ^7^IRMB, University of Montpellier, INSERM, CNRS, CHU Montpellier, Montpellier, France

**Keywords:** polyoxidinidum, dendritic cells, cytotoxic lymphocyte, breast cancer, aliphatic polyamines, natural killer cells, T cell

## Abstract

Immunotherapy, which is seen as a major tool for cancer treatment, requires, in some cases, the presence of several agents to maximize its effects. Adjuvants can enhance the effect of other agents. However, despite their long-time use, only a few adjuvants are licensed today, and their use in cancer treatment is rare. Azoximer bromide, marketed under the trade name Polyoxidonium® (PO), is a copolymer of N-oxidized 1,4-ethylenepiperazine and (N-carboxyethyl)-1,4-ethylene piperazinium bromide. It has been described as an immune adjuvant and immunomodulator that is clinically used with excellent tolerance. PO is used in the treatment and prophylaxis of diseases connected with damage to the immune system, and there is interest in testing it in antitumor therapy. We show here that PO treatment for 1 week induced positive pathological changes in 6 out of 20 patients with breast cancer, including complete response in a triple-negative patient. This correlated with an increased tumor CD4^+^ T-lymphocyte infiltration. The immune effects of PO are associated with myeloid cell activation, and little is known about the action of PO on lymphocyte lineages, such as natural killer (NK) and T cells. We reveal that PO increases T-cell proliferation *in vitro* without negative effects on any activation marker. PO does not affect dendritic cell (DC) viability and increases the expansion of immature DC (iDC) and mature DC (mDC) at 100 μg/ml, and it stimulates expression of several DC co-stimulatory molecules, inducing the proliferation of allogeneic T cells. In contrast, PO decreases DC viability when added at day 5 post-expansion. PO is not toxic for NK cells at doses up to 100 μM and does not affect their activation, maturation, and cytotoxicity but tends to increase degranulation. This could be beneficial against target cells that show low sensitivity to NK cells, e.g., solid tumor cells. Finally, we have found great variability in PO response between donors. In summary, our *in vitro* results show that PO increases the number of costimulatory molecules on DC that prime T cells, favoring the production of effector T cells. This may support the future clinical development of PO in cancer treatment.

## Introduction

Immunotherapy is now seen as the new frontier for cancer treatment, with several impressive successes (1). In several cases, cancer immunotherapy requires that immune responses be targeted toward specific antigens, and there is usually a lack of efficient stimulation of the immune system. In this sense adjuvants, agents that modify the effect of other agents, boost the immune response and have been largely used in vaccination (2), thus minimizing the dose of antigen needed. Despite their long-time use, only a few adjuvants are licensed today to generate an adaptive immune response to vaccines, and their use in cancer treatment is rare (3).

Polyoxidonium (PO) is a physiologically active compound from a new class of heterochain aliphatic polyamines that are attracting clinical interest (4). Chemically, PO is a copolymer of N-oxidized 1,4-ethylenepiperazine and (N-carboxyethyl)-1,4-ethylene piperazinium bromide, which is soluble in water and biodegradable and has a molecular weight of 60–100 kD (5). PO is approved in Russia as a vaccine adjuvant drug that stimulates antibody production (http://petrovax.com/medication/catalog/polyoxydonium/). The copolymer chains are cleaved and easily released from the body (5), which explains its low renal toxicity and good safety profile, as demonstrated in an extensive post-marketing study in Slovakia (6). Correspondingly, PO complexed with antigens in commercial influenza vaccine has also demonstrated high safety according to an analysis of about 50 million recipients (5).

PO binds to human peripheral blood monocytes and neutrophils, and, to a lesser extent, to lymphocytes (7). It is used as an immune adjuvant, particularly for vaccines, and as an immune modulator for the treatment of acute and chronic bacterial, viral, or fungal infectious diseases (4). PO has several immunogeneic properties. First, it stimulates the production of IL-6 (7). Second, it increases the bactericidal activity of leukocytes (8). Third, PO induces H_2_O_2_ production and improves the capacity of neutrophils and macrophages to capture and process different infectious agents, including bacteria, e.g., staphylococci, by about 40–60% (7, 8). This can explain PO's ability to enhance resistance to infections.

The effect of PO on the lymphocytic compartment is less known, although its immune-modulatory functions could partially involve improved antigen presentation resulting in effective antibody production (4). First, PO was covalently conjugated to antigenic components of the influenza vaccine: hemagglutinin and neuraminidase (5). Data from about 50 million recipients indicated that the vaccine was safe and effective (5). Second, PO was evaluated with trivalent live attenuated measles, mumps and rubella vaccine (9). Healthy children did not need PO to produce a high level of specific antibodies. In contrast, children with abnormal T cell counts could benefit from the use of PO (9).

Due to the excellent clinical safety profile of PO, we decided to try it in breast cancer patients prior to surgery. We observed a positive clinical outcome in 30% of the patients, which correlated with increased tumor infiltration by CD4^+^ T cells. The results prompted us to investigate the effects of PO *in vitro* to identify cell targets on three different immune lineages playing important roles in tumor immune surveillance, namely dendritic cells (DC), T-cells, and NK cells (10). We found, however, that several immunomodulatory properties of PO varied between donors. Hence, there is a real need for a better understanding of the immune effects of PO to support new clinical developments.

## Patients, Materials, and Methods

### Compounds

PO was provided by NPO Petrovax (Moscow, Russia). Recombinant human (rh) IL-15 obtained from Miltenyi and rhIL-2 from PeproTech. Recombinant human GM-CSF and rhIL-4 were obtained from R&D systems and LPS from Sigma. All other products are described below.

### Breast Cancer Patients

PO is authorized in Russia and in other countries as an immune adjuvant. Patients were treated in the department of surgery at the N.N. Blokhin National Oncology Research Center in Moscow according to the internationally approved guidelines and regulations used by the local Ethics Committee. Pathologists morphologically verified the presence of cancer by staining with hematoxylin-eosin before PO treatment. Twenty patients with histologically confirmed breast adenocarcinoma without metastasis received neoadjuvant PO at a dose of 12 mg by intramuscular injection at days 1, 2, 3, 5, and 7. Staging was determined using the TNM classification (11). Table 1 describes the patients' stages; according to this classification, T describes the size of the original (primary) tumor and whether it has invaded nearby tissue, N describes nearby (regional) lymph nodes that are involved, and M describes distant metastasis. We also analyzed Her2/neu, the estrogen and progesterone receptors, and Ki-67 as a proliferative index. Patients had subsequent surgery at day 8. Pre- and post-surgery pathological samples were compared according to a pathomorphosis scoring system that defines the pathological changes observed between samples performed before and after a specific therapy, as previously described (12, 13). Briefly, pathomorphosis degree 1 corresponds to mild modification, degrees 2 and 3 correspond to low to moderate reduction of tumor cell infiltrate, and degree 4 indicates complete disappearance of the tumor cell infiltrate. We also studied the subsets of leucocytes infiltrating the tumor and, moreover, we analyzed the changes in lymphocytes in blood and in bone marrow aspirates at Day 0 and Day 8 in nine patients. Cell suspensions were analyzed for CD4/CD3/CD25/CD45 and CD8/CD3/CD56/CD45 using Flow Cytometry and the FCS3 program (Becton Dickinson, Bioline BD Biosciences, St. Petersburg Russia).

**Table 1:** Clinical characteristics of the 20 breast cancer patients treated with PO.

Number of patients	20
Median age (range)	53.5 years (32–78)
**TNM**	
T1	1
T2	19
N0	6
N1	7
N2	7
N3	0
M0	20
**Histological pattern**	
Infiltrative ductal carcinoma	14
Infiltrative lobular	4
Tubular	1
Medullar	1

*The table describes patient stages; according to the TNM classification, T describes the size of the original (primary) tumor and whether it has invaded nearby tissue, N describes nearby (regional) lymph nodes that are involved, and M describes distant metastasis. Histological pattern and the pathological response are also shown*.

### Healthy Donor Samples

Data were obtained from three individual donors of the “Etablissement Français du Sang” (*EFS*). We prepared three biological samples from each buffy coat for each of the following experiments: PO concentration and cell type. This work benefited from umbilical cord blood units (UCBs) and the expertise of Prof. John De Vos, in charge of the Biological Resource Center Collection of the University Hospital of Montpellier–http://www.chu-montpellier.fr/en/platforms (BIOBANQUES Identifier–BB-0033-00031).

### *In vitro* Dendritic Cell (DC) Expansion/Differentiation/Maturation

After Ficoll purification, PBMCs were plated in RPMI 1640 Medium supplemented with 10% fetal calf serum, 100 U/ml penicillin, 0.1 mg/ml streptomycin, and 1% glutamine (RP10), and 2 h later, non-adherent cells were removed. Adherent cells were used as a starting population and cultured in RP10 medium supplemented with rhGM-CSF (100 ng/ml) and rhIL-4 (25 ng/ml) for 7 days.

PO was dissolved in water and added to cells growing in RP10 media at several final concentrations (1, 10, or 100 μg/ml). PO was added from day 0 to day 7 (immature DC, iDC D0). We added 1 ml of fresh RP10 medium supplemented with GM-CSF and IL-4 at day 2. At day 5, some iDCs (iDC D5) were treated with various concentrations of PO (1, 10, and 100 μg/ml) to examine its effect on iDC maturation. As a positive control, we used LPS (50 ng/ml) to induce DC maturation (mDC), and we also investigated the effects of PO on the LPS-induced maturation. Experiments were performed in triplicate, and the results are expressed as mean ± SEM. The expression of cell surface molecules was analyzed after 7 days of culture. Cell staining was performed using fluorescent-conjugated monoclonal antibodies. The antibodies PE-Cy™5-conjugated anti CD1a (clone HI149), BV421-conjugated anti CD83 (clone HB15e), BV605-conjugated anti CD80 (clone L307.4), BV650-conjugated anti CD14 (clone M5E2), BV711-conjugated anti CD40 (clone 5C3), PE-Cy™7-conjugated anti CD86 (clone 2331), Alexa Fluor®647-conjugated anti CCR1 (clone 53504), and Fc-Block were obtained from BD Biosciences. The APC-Alexa Fluor®750-conjugated anti-HLA-DR antibody (clone Immu-357) was purchased from Beckman Coulter. The Human CCR7 Fluorescein-conjugated Antibody (clone 150503) was purchased from R&D Systems, and phycoerythrin (PE)-conjugated anti CD1c (clone AD5-8E7) was purchased from Miltenyi Biotech. Samples were acquired on a BD-LSR Fortessa (Becton Dickinson), and all data were analyzed using FlowJo software (Tree Star, Ashland, OR, USA). iDCs were identified by expression of CD1a and CD1c and loss of CD14 expression, and DC maturation was monitored by expression of CD40, CD80, CD83, and CD86 in the HLA-DR+ cells.

### *In vitro* T-Cell Expansion/Activation

Cells were treated with PO all through the expansion/activation protocol. Several concentrations were used: PO 1, 10, 100, and 500 μg/ml. After Ficoll, we used the EasySep™ CD3 positive selection kit (StemCell Technologies) according to the manufacturer's protocol. Purified CD3^+^ cells were resuspended in RPMI-Glutamax, 10% FBS. Cells were activated by human T-activator CD3/CD28 Dynabeads (Life Technologies) according to the manufacturer's protocol. At day 5, we analyzed T-cell proliferation and different T-cell markers. Some samples were treated with PMA (50 ng/ml)/Ionomycin (1 μg/ml). Intracellular staining was performed by adding BD GolgiPlug, a protein transport inhibitor containing Brefeldin A (BD Biosciences).

The expression of cell surface molecules was analyzed after 7 days of culture. Cell staining was performed using fluorescent conjugated murine monoclonal antibodies. V500-conjugated anti CD4 (clone L200), PerCP-Cy5.5-conjugated anti CD25 (clone MA251), V450-conjugated anti Foxp3 (clone 259D/C7), PE-conjugated anti CD127 (clone HIL7RM21), PE-Cy7-conjugated anti IFN-g (clone B27), and PerCP-Cy5.5-conjugated anti IL-17a antibodies were obtained from BD Biosciences. PB-conjugated anti CD4 (clone 13B8.2) and APC-conjugated anti CD8 (clone B9.11) were purchased from Beckman Coulter. Stained samples were analyzed on a Gallios flow cytometer (Beckman Coulter) using Kaluza software. The experiment was performed in triplicate, and the results are expressed as mean ± SEM.

### Expansion and Activation of Human NK Cells

This was performed as previously described (14). Briefly, blood units were depleted of T cells by using an EasySep^TM^ CD3 Positive Selection Kit (STEMCELL Technologies). Cells were cultured for 20 days with γ-irradiated PLH cells at a 1:1 NK cell:accessory cell ratio in the presence of IL-2 (100 U/mL) and IL-15 (5 ng/mL). PLH cells were added every 4 days and fresh cytokines every second day. We did not add PLH for the last 4 days, and, at the end of the process, NK cell purity (CD56^+^/CD3^−^) was always higher than 90% with no living PLH cells remaining.

For phenotype analysis, cells were stained with 7AAD (Beckman Coulter) to identify viable cells and antibodies against surface markers. FITC-conjugated anti CD25 (clone B1.49.9), anti CD45RO (clone UCHL1), PE-conjugated anti CD69 (clone TP1.55.3), anti CD62L (clone DREG56), anti CD19 (clone J3-119), anti CD3 (clone UCHT1), ECD-conjugated anti CD19 (clone J3-119), PacificBlue-conjugated anti CD16 (clone 3G8), anti CD57 (clone NC1), APC-AlexaFluor750-conjugated anti CD45 (clone J33), anti CD45RA (clone 2H4LDH11LDB9), KromeOrange-conjugated anti CD45 (clone J33), and anti CD16 (clone3G8) were obtained from Beckman Coulter. FITC-conjugated anti CD158b (clone CH-L), PE-conjugated anti CD158a (clone HP-3E4), and V450-conjugated anti CD107a (clone H4A3) were provided by BD Biosciences. APC-conjugated anti CD56 (clone REA196), anti CD3 (clone AC146), and Vioblue-conjugated anti CD158e (clone DX9) were purchased from Miltenyi. PECy7-conjugated anti CD56 (clone HCD56) was obtained from BioLegend. 1 × 10^5^-3 × 10^5^ cells were incubated for 20–30 min at 4°C with different antibodies in PBS containing 2.5% FBS. Cells were then washed and suspended in 200–250 μL of the same media. Stained samples were analyzed on a Gallios flow cytometer (Beckman Coulter) using Kaluza software. Viable lymphocytes were gated using FSC/SSC and 7AAD staining. B cells (CD19^+^), T cells (CD3^+^CD56^−^), and NK cells (CD56^+^CD3^−^) were distinguished using CD19, CD3, and CD56 antibodies, respectively.

### NK Cell-Mediated Cytotoxicity

This was performed as previously described (14, 15). NK cells were labeled with 3 μM of CellTracker™ Violet BMQC Dye (Life Technologies) and incubated overnight with target cells at different E:T ratios. Subsequently, phosphatidylserine (PS) translocation and membrane damage were analyzed in the violet fluorescence-negative target cell population by flow cytometry using Annexin V-FITC (Immunostep) and 7AAD (BD Biosciences) or propidium iodide (PI) as previously described (16, 17). We consider all cells positive for annexin-V and/or PI (or 7-ADD) as dead (or dying).

### NK Degranulation Assay

This was done as previously described (15). Briefly, 50 × 10^3^ target cells per well were placed in RPMI, 10% FBS, IL-2 100 U/mL with monensin (BD Biosciences) in a 96-well V-bottom plate. NK and target cells were incubated overnight at 37°C in 5% CO_2_, and living cells were counted using a Muse cytometer (Millipore) with a count and viability kit (Millipore). As a control, NK cells were incubated without target cells. CD107a^+^ NK cells were analyzed on a Gallios flow cytometer (Beckman Coulter) using 7AAD, CD45RO-FITC, CD19-PE, CD56-PECy7, CD3-APC, CD45RA-APCAlexaFluor750, CD16-KromeOrange, and CD107a-HV500 (BD Biosciences). The results were analyzed using Kaluza software.

### Statistical Analysis

Experimental values were processed and statistical analysis was performed using GraphPad Prism (v6.0) software. All statistical data are provided as *p < 0.05; **p < 0.01; ***p < 0.001, and ****p < 0.0001. Mean values are expressed as mean plus or minus the standard error of the mean (SEM).

Results were obtained from three individual donors of the “Etablissement français du sang” (*EFS*). We prepared three biological samples from each buffy coat for each of the following experiments: PO concentration and cell type.

## Results

### Effects of PO in Patients With Breast Cancer

**Figure 1:**
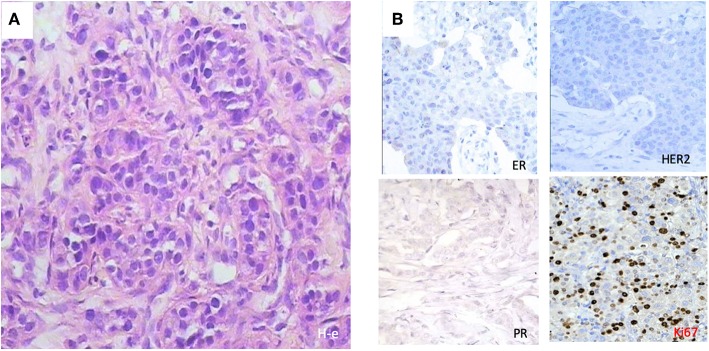
Histological patterns at diagnosis of a 32-year-old patient with triple negativity that reached a complete pathological response after PO treatment. **(A)** Morphological features with hematoxylin-eosin (H-e) staining (x50) showed ductal tumor infiltrate. **(B)** Immunohistochemical stainings showed triple negativity for progesterone receptor (PR), estrogen receptor (ER), and human estrogen growth receptor 2 (HER2), with a high Ki-67 index.

**Figure 2:**
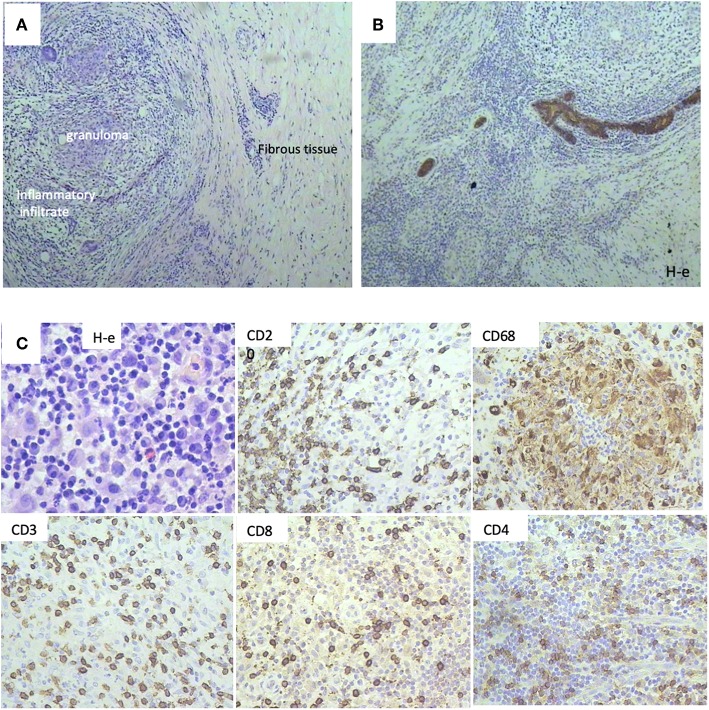
Histological patterns after Polyoxidonium (PO) administration of a 32-year-old patient with triple negativity that reached a complete pathological response after treatment. **(A)** Morphological aspects, with the presence of granuloma, inflammatory infiltrate, and tissue fibrosis, with no tumor cell infiltrate (H-e; x20). **(B)** Immunohistochemical study with pancytokeratin antibodies (AE1/AE3) followed by Mayer haematoxilin staining. Positivity was only limited to breast ducts with no tumor cell infiltrate, corresponding to a complete pathological response. **(C)** Immune infiltrate was observed in the biopsy sections, with immunohistochemical staining on post-surgical material. T-lymphocytes (CD3, CD4, CD8 positive cells), B-lymphocytes (CD20 positive cells), and macrophages (CD68 positive cells) were observed.

We selected a cohort of 20 female patients with a mean age of 53.5 years (range 32–78). The first biopsy at day O showed that they had diverse TNM scores and that the majority had infiltrative ductal carcinoma (Table 1). We treated patients intramuscularly with PO for 1 week and obtained a second biopsy at day 8. Among the patients, six demonstrated pathological changes after surgery compared to results before surgery (Table 2). Responding patients had diverse TNM scores, estrogen and progesterone receptors, Her2/neu staining, and proliferative indexes (Table 2). One patient had the particularly aggressive triple-negative phenotype, i.e., negative for the receptors for estrogen, progesterone, and Human Epidermal Growth Factor 2 (HER2), and had a high proliferative index as measured by a high proliferation ratio based on Ki-67 staining (Figure 1). She received 1 week of PO treatment and received a radical mastectomy of the left breast with saving of breast muscles. We observed pathomorphosis degree 4 in the post-surgery material (Figures 2A,B). This corresponds to a total disappearance of the tumor cell infiltrate, meaning complete pathological response. We also observed an inflammatory infiltrate in the lodge of the tumor (Figure 2C), which was composed of cells of both cellular and humoral immunity with an almost equivalent ratio of T and B cells (Figure 2C). Macrophages were composed of separate histiocytes, clustered as granulomas, and showed the appearance of a giant cell reaction (Figure 2C). Finally, around 15–20% of infiltrating cells were granulocytes, mainly neutrophils (data not shown). Hence, the changes detected after PO treatment in this patient corresponded to active chronic inflammation with granuloma formation.

**Table 2:** Clinical characterization of patients with pathomorphosis after PO treatment (up) and receptor status and proliferative index of their tumors (down).

**Pt number**	**Stage**	**pT**	**N**	**Pathomorphosis degree**
1	IIb	2	1	4
2	IIIa	2	2	1
3	IIIa	2	1	1
4	IIb	2	0	2
5	I	1	0	1
6	IIIa	2	2	2
**Pt number**	**Estrogen receptors**	**Progesterone receptors**	**Her2/neu**	**Ki-67**
1	0	0	0	75
2	7	5	1	28
3	3	5	0	18
4	7	0	3	31
5	7	6	0	26
6	0	0	2	20

*The table describes patient stages; according to the TNM classification, T describes the size of the original (primary) tumor and whether it has invaded nearby tissue and N describes nearby (regional) lymph nodes that are involved. Numbers under estrogen and progesterone receptors mean the standard score of receptor positivity in points. Her2/neu is given as a score, which is in points from 0 (negative) to 3 (positive). Ki-67 is a proliferative index in percentage of positive cells*.

In addition, minor changes (degree 1) were observed in three patients, and a reduction of the tumor infiltrate (degree 2) was found in two patients. We also obtained cell suspensions and studied the subsets of leucocytes infiltrating the tumor. We found a significant difference in the CD4^+^ population on the tumor cell infiltrate between the six patients with pathological changes and those without pathological changes, respectively 50.91 ± 2.05% vs. 40.89 ± 2.26% (*P* = 0.006). In addition, the CD8/CD4 ratio was 0.79 ± 0.09 vs. 1.17 ± 0.13 (*P* = 0.03). We also analyzed the CD4 and CD8 subsets in blood and in bone marrow aspirates of nine of our patients at day 0 and at day 8 after PO treatment and did not find any significative difference (data not shown).

### Effects of PO in T Cells

**Figure 3:**
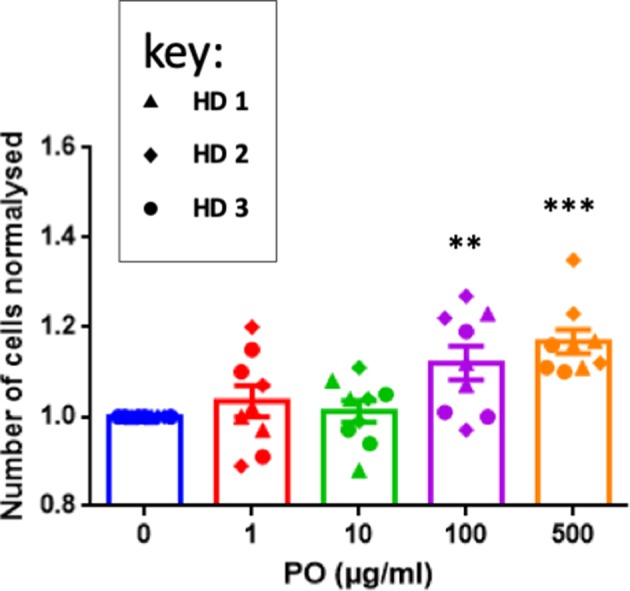
PO increases T-cell proliferation. Purified T cells from three individual healthy donors were activated by anti-CD3/CD28 costimulation in triplicate. The indicated concentrations of PO were added at Day 0. The number of cells was quantified. Control non-treated cell average count was standardized to 1 to decrease the variability between donors. Graphs represent means ± SEM; **p < 0.01, ***p < 0.001; ANOVA test was used for comparison to non-treated cells.

The previous results concerning T-cell recruitment prompted us to investigate direct effects of PO on T cells. We stimulated purified T cells from three healthy donors (HD) with anti-CD3/CD28 antibodies. This protocol induced efficient activation, as measured by CD127 downregulation and CD25 upregulation (Supplementary Figure 1A) and CD45RA downregulation (data not shown). To partially mimic the relatively long-term PO treatment in breast cancer patients, we stimulated T cells and treated them with different PO concentrations. T cell counts statistically increased by day 5 at 100 and at 500 μg/ml (Figure 3). In contrast, PO did not change the percentage of CD4^+^ cells expressing CD127, CD25, and CD45 nor their level of expression (Supplementary Figure 2). Similarly, PO did not affect CD8^+^ activation, except that decreased CD25 levels were induced by higher PO doses (Supplementary Figure 3). T-cell restimulation at day 5 with PMA/ionomycin induced IFNγ production in both T-cell compartments. This response was not affected by PO (Supplementary Figure 3). In summary, *in vitro* chronic PO treatment had no toxic effect and, in fact, increased T-cell proliferation without negative effects on any of the activation markers tested.

### Effects of PO on Dendritic Cells (DCs)

DCs are key regulators of immune responses, capable of priming naive resting T cells and initiating primary T-cell responses. Hence, the effects of PO on T cells in breast cancer patients could be mediated by DC modulation. We treated PBMC adherent cells with granulocyte-macrophage colony-stimulating factor (GM-CSF) and interleukin-4 (IL-4) for 5 days to generate immature DCs (iDCs) and promoted their maturation (mDCs) by an additional 2-day incubation with lipopolysaccharide (LPS). We analyzed the effect of PO on DC generation and maturation using three protocols. Firstly, we added several PO concentrations from day 0 to 7. This indicates the capacity of PO to influence the generation/expansion of iDCs. Secondly, we treated iDCs with PO at day 5 (D5). This allowed us to monitor PO ability to induce iDC maturation *per se*, therefore indicating if PO favors the formation of a humoral and/or cell-mediated immune response. Thirdly, we maturated iDCs with LPS while at the same time treating cells with several PO concentrations. This examines the ability of PO to affect LPS-induced DC maturation. iDCs were identified by CD1a and CD1c expression and loss of CD14 expression. DC maturation was monitored by expression of the co-stimulatory molecules CD40, CD80, CD83, and CD86 in HLA-DR+ cells. The percentage of iDCs that expressed the costimulatory molecules CD80, CD86, CD40, and CD83 was relatively low and increased significantly in LPS-induced mDCs (Supplementary Figure 4).

**Figure 4:**
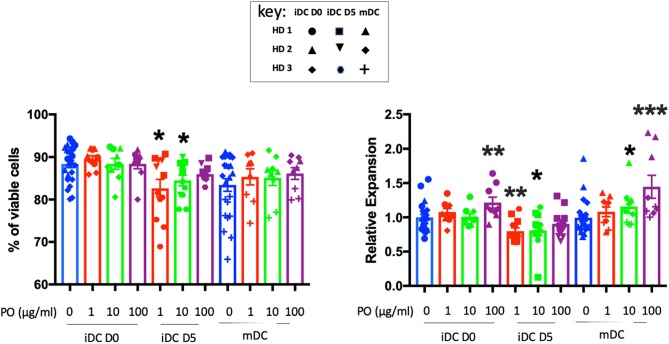
Effects of PO on DC viability and expansion. Number of cells (right) and their viability (left) obtained at Day 7 from three individual healthy donors. Various concentrations of PO (1, 10, and 100 μg/ml) were added either at Day 0 (iDCs D0) or Day 5 (iDCs D5). DC maturation (mDCs) was induced by the addition of LPS (50 ng/ml) in the absence or presence of various PO concentrations. Due to inter-donor variability, we normalized expansion, giving the value of 1 to the iDC expansion and adjusting other values to that number. Graphs represent means ± SEM; *p < 0.05, **p < 0.01, ***p < 0.001; Mann–Whitney U was used for comparison to non-treated cells.

PO did not affect iDC and mDC viability and increased iDC and mDC generation when added at 100 μg/ml at day 0 (Figure 4). In contrast, when added at day 5 at 1 and 10 μg/ml, it decreased DC viability slightly (for HD3) and, consequently, the final count (Figure 4). We observed variability between donors, but PO was consistently well tolerated in terms of DC viability and expansion (Supplementary Figure 5). Taken together, these results suggested that PO could improve the expansion of mDCs. Hence, in sites of inflammation sites where DC maturation occurs, PO would facilitate the production of mDCs.

**Figure 5:**
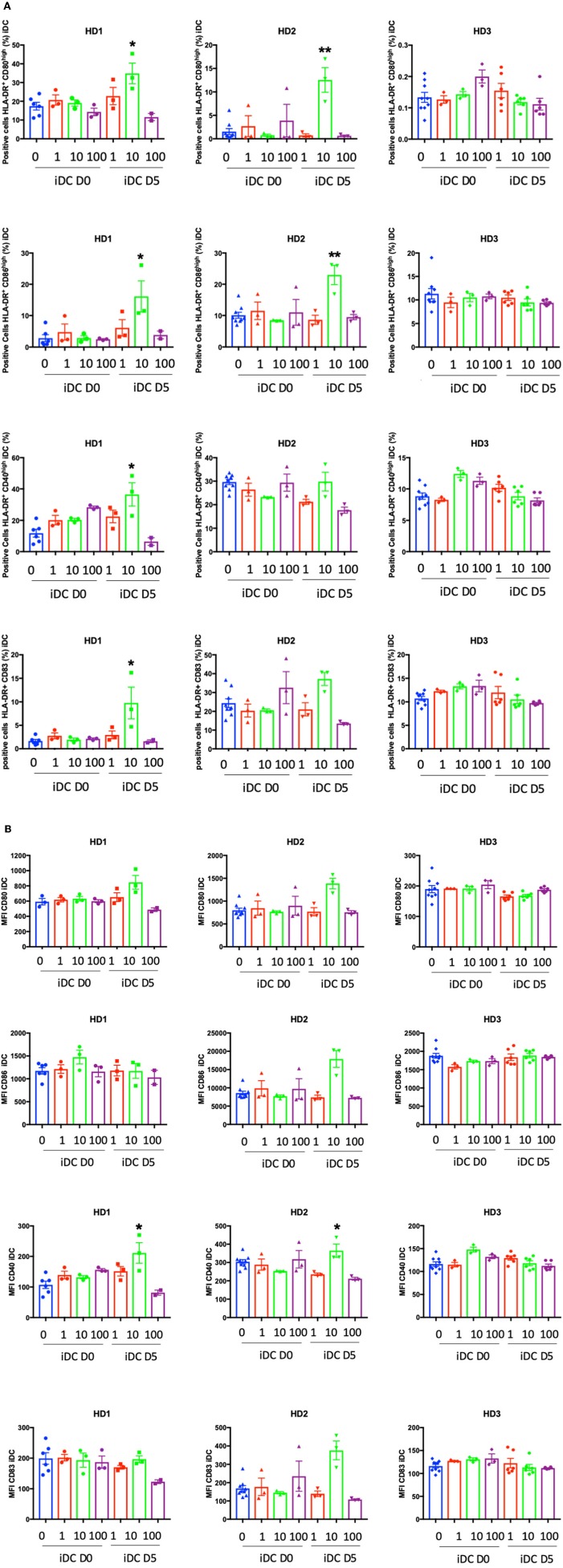
Effect of PO on the percentage of DCs expressing DC maturation markers. iDCs were induced as described in the caption of Figure 4. **(A)** Percentage of DCs expressing various markers. **(B)** The expression levels (MFI) of DC maturation markers were analyzed. Graphs represent means ± SEM of each individual healthy donor with biological experiments performed in biological triplicates. *p < 0.05, **p < 0.01; Mann–Whitney U test was used for comparison to non-treated cells.

Next, we performed phenotypic analyses at D7 to evaluate the level of expression of HLA-class II and the co-stimulatory molecules CD40, CD80, CD83, and CD86 (Supplementary Figure 4). The expression of these surface markers increases during DC maturation and could be revealing regarding the immunogenic properties of PO. PO did not affect the expression of any of these markers during iDC generation in terms of the percentage of positive cells (Figure 5A) or the expression levels (Figure 5B). The addition of 10 μg/ml PO at day 5 increased the percentage of iDCs expressing some costimulatory molecules in two out of three donors (Figure 5A). Moreover, the same PO concentration increased the expression levels of some costimulatory molecules in these patients (Figure 5B). This shows that PO has immunogenic properties. PO did not change LPS-induced expression of costimulatory molecules (Supplementary Figures 4, 6). In fact, the LPS-induced increase was already very high, suggesting that PO is not capable of increasing it further.

Finally, to validate the PO-induced DC maturation, PO-treated DCs were co-cultured with CFSE-labeled T cells to evaluate their ability to induce allogeneic CD4 and CD8 T-cell proliferation. We used immature and mature DCs as controls. Firstly, it is important to note that both CD4 and CD8 T-cell proliferation was induced in all conditions tested in the three independent biological experiments for two individual donors.

**Figure 6:**
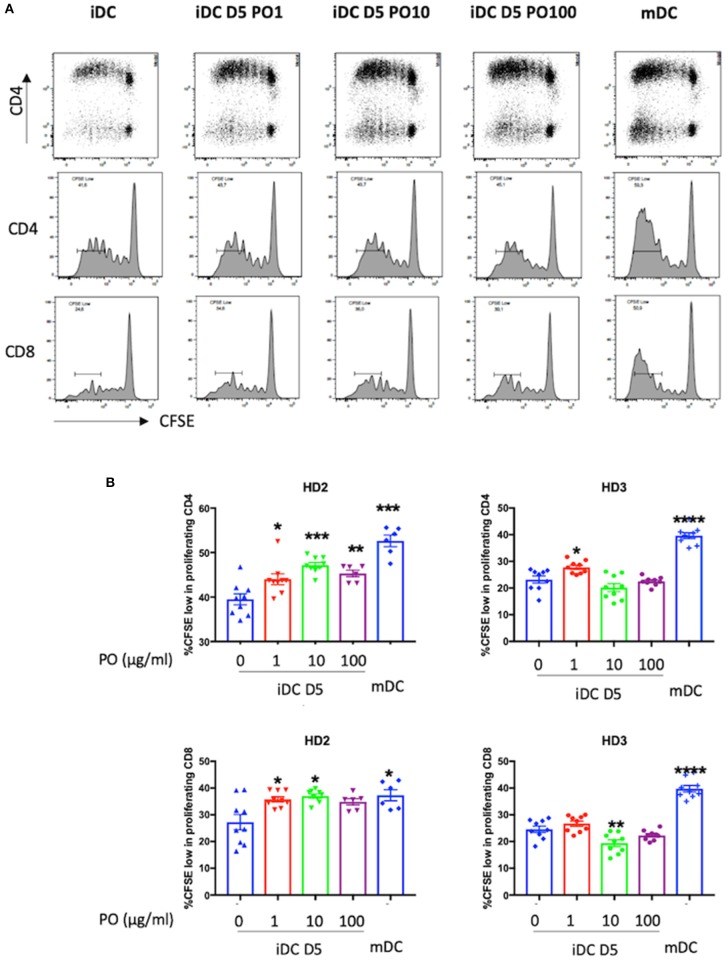
PO increased iDC potential to stimulate mixed lymphocyte reaction (MLR). iDCs treated at D5 with different concentrations of PO (0, 1, 10, 100 μg/ml) or mDCs, as described in the caption of Figure 4, were used to stimulate CFSE-labeled allogeneic T-cells at a DC:T-cell ratio of 1:40. **(A)** Proliferation of T cells was analyzed at day 5. All conditions were tested at least in triplicate. Figure presents representative dot plots (top) and histograms depicting the proliferation of allogeneic CFSE-labeled CD4^+^ (middle) and CFSE-labeled CD8^+^ cells (bottom). **(B)** Percentage of highly proliferating CD4 (top) and CD8 cells (bottom). *p < 0.05, **p < 0.01, ***p < 0.001, ****p < 0.0005; Mann–Whitney *U* was used for comparison to non-treated cells.

Higher proliferating T cell counts were observed following incubation with mDCs in both donors. Incubation with PO-treated iDCs increased the proliferation of allogeneic CD4 and CD8 T cells compared with control iDCs (Figure 6). Although these results varied between donors, they demonstrated the good immunogenicity of PO-treated DCs and their potential to induce CD4 and CD8 T-cell proliferation.

In conclusion, PO does not affect DC differentiation but may promote their maturation and the expression of co-stimulatory molecules leading to good DC immunogenicity reflected by T-cell proliferation. However, there is variability, suggesting that the effects of PO could depend on patient/donor immune status.

### Effects of PO in NK Cells

NK cells are parts of the innate immune system and have natural cytotoxicity. NK cells predominantly target cells lacking MHC-I, including transformed or virus-infected cells, which downregulate MHC-I expression to avoid recognition by CTLs. Therefore, the “missing self” hypothesis proposes that NK cells discriminate target cells from other healthy “self” cells based on MHC-I expression. However, it is now clear that NK-cell activation depends on a complex signaling process mediated by activating and inhibitory receptors. The outcome depends on the strength of the various activating and inhibitory signals. The inhibitory receptors mainly recognize MHC-I (HLA in humans) molecules, and activating receptors can recognize stress ligands in target cells. Therefore, NK cells also eliminate “stressed” cells even if they express normal MHC-I levels.

**Figure 7:**
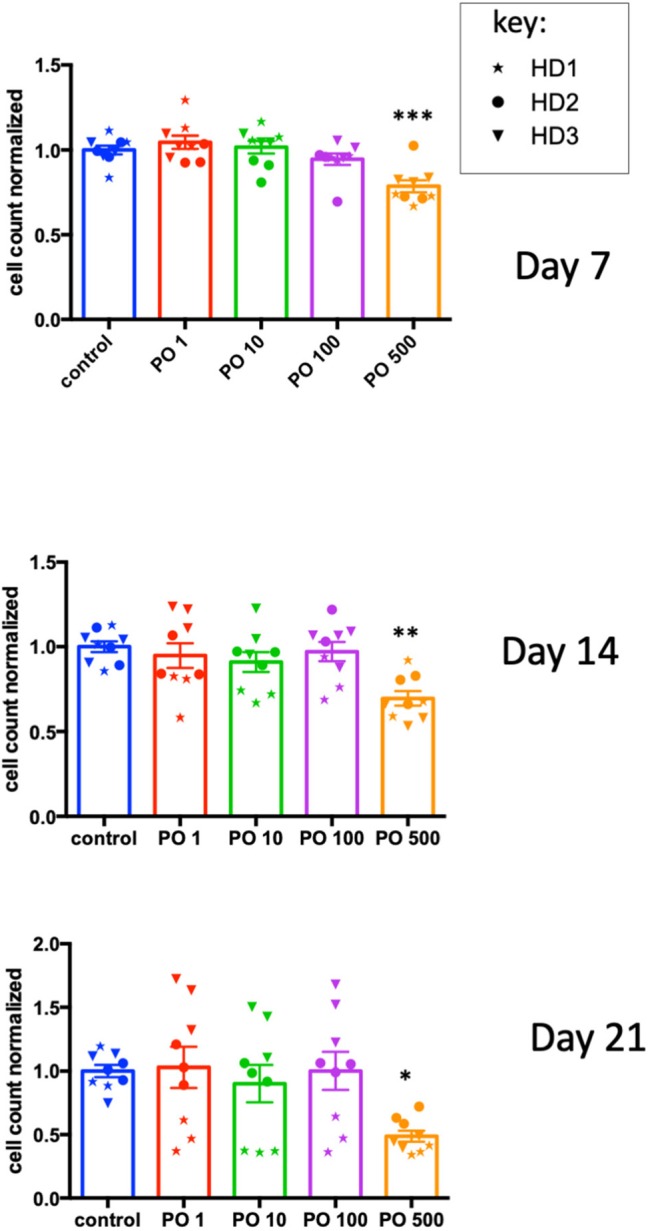
High PO concentration decreases NK cell proliferation. NK cells from three individual healthy donors were activated by costimulation with target cells and low doses of cytokines for different times periods. Various concentrations of PO were added at day 0, and the number of cells quantified. The average control values were standardized to 1 to decrease the variability between donors. Graphs represent means ± SEM; *p < 0.05, **p < 0.01, ***p < 0.001; ANOVA test was used for comparison to non-treated cells.

As NK cells provide anticancer defense (18, 19), we analyzed the effects of PO on NK-cell activation and expansion. CD3^+^-depleted PBMC cells were incubated at different PO concentrations, and the cell number was analyzed at day 7, 14, and 21. We observed decreased counts only at 500 μg/ml concentrations, but the effect was not statistically different at any concentration (Supplementary Figure 7). In fact, the raw data were very heterogeneous due to differences in the initial NK cell numbers in the blood bags and the response to the activation/expansion protocol. This was expected, given the variable percentages of NK cells in healthy humans (roughly 5–20%) and the NK cell responses to different stimuli (14). To counteract this issue, we calculated the average cell counts for each donor and produced a normalized value. We used this to normalize values in PO-treated cells. This showed that PO at 500 μg/ml decreased NK cell proliferation (Figure 7). In contrast, lower concentrations did not produce any effect. Therefore, PO does not affect NK-cell viability and proliferation at concentrations below 100 μg/ml and up to 21 days of stimulation, suggesting that these concentrations should not negatively affect NK-cell viability and proliferation in patients. In this context, it should be noted that peripheral NK cells have a short lifespan, 1 week on average (20), so it is unlikely that most NK cells will be in contact with PO for longer periods.

We next investigated the effects of PO on three well-known NK-cell activation markers, CD69, CD25, and the transferrin receptor CD71, at day 21 post-activation (Supplementary Figure 8A). CD71 is upregulated in cells with high metabolism. CD25 is the high affinity IL-2 receptor. CD69 is an early activation NK marker. All these markers were increased after NK-cell activation, and their expression was higher in NK with high anticancer activity (21, 22). None of the PO concentrations affected the percentage of NK cells expressing CD69 and CD71 or their expression levels as measured by MFI intensities. We observed a trend toward an increased percentage of CD25 positive cells and higher CD25 levels at PO 10 μg/ml. However, generally, PO did not change the expression of these activation markers.

During NK-cell maturation, CD56^bright^ cells become CD56^dim^CD62L^+^CD57^−^ cells, which produce perforin while maintaining high IFN-γ production in response to cytokines (23, 24). CD56^dim^CD62L^−^CD57^+^ cells then show low response to cytokines and higher cytotoxic capacity and are considered fully mature NK cells (23, 25). *In vitro* stimuli for periods of up to 20 days do not induce CD57 (14). PO at 10–100 μg/ml showed a tendency to decrease the expression of both markers (Supplementary Figure 8B). Finally, fully mature, cytotoxic, NK cells express inhibitory Killer-cell immunoglobulin-like receptors (KIRs) and CD16 (26). Supplementary Figure 8B shows a trend toward an increase in KIR expression and no changes in CD16 in PO-treated cells (up to 100 μM).

Cytotoxic NK-cell function is mediated by activating receptors, e.g., NKG2D, which recognizes stress ligands in target cells. Their engagement induces natural cytotoxicity. In addition, NK cells recognize Fc domains in mAb-opsonized targets by the FcγRIIIa (CD16a). The engagement induces antibody-dependent cell-mediated cytotoxicity (ADCC). PO at doses below 500 μM did not affect the expression of these essential receptors (Supplementary Figure 8B).

CD45 is a protein, tyrosine phosphatase, that is specifically expressed in leucocytes (27). The largest isoform, CD45RA, is expressed on naïve T cells. Activated and memory T lymphocytes express the shortest CD45 isoform, CD45RO, which lacks RA, RB, and RC exons. This shortest isoform facilitates T-cell activation. The expression of CD45 isoforms gives NK cells different functional properties (22). NK cells coexpressing the long (CD45RA) together with the short (CD45RO) isoforms show higher antitumor activity in hematological cancer patients (21). After *in vitro* stimulation, CD45RA+ are resting cells, CD45RO+ are activated, CD45RARO show higher cytotoxicity, and CD45RAdim are cells in the process of activation. PO did not significantly change these *in vitro*-generated populations (Supplementary Figure 9), although there was a trend toward decreased expression of CD45RA and toward increased CD45RO, which could represent increased cytolytic activity (22).

**Figure 8:**
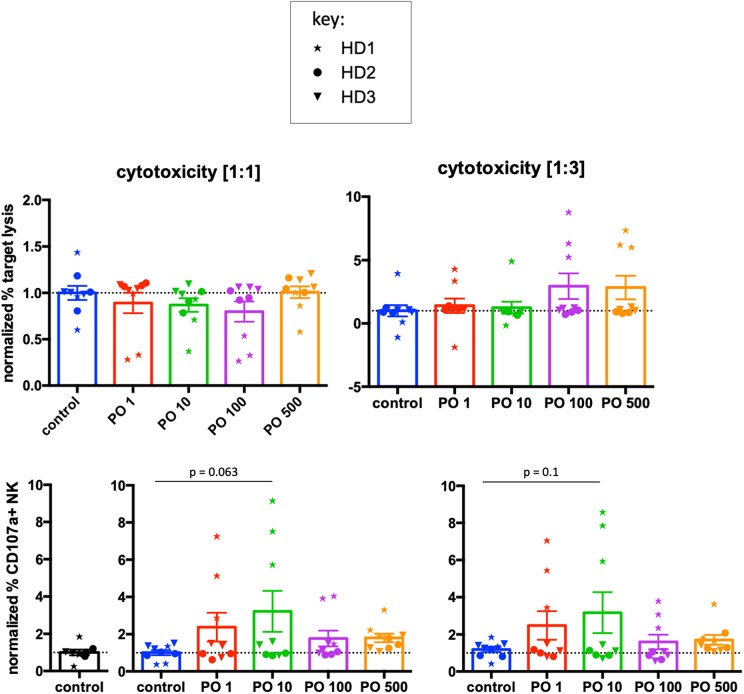
PO does not affect NK-cell cytolytic function. NK cells from three individual healthy donors were activated by costimulation with target cells and low doses of cytokines for 21 days. Various concentrations of PO were added at day 0. Upper graphs represent toxicity against tumor cells from a B cell lymphoma patient at two different effector:target (E:T) ratios. Lower graphs represent the percentage of CD107^+^ cells. The averages of the control values were standardized to 1 to decrease the variability between donors. Graphs represent means ± SEM; ANOVA test was used for comparison to non-treated cells.

We next analyzed the effects of PO on NK-cell-mediated cytotoxic function by investigating natural cytotoxicity and degranulation. We used two effector:target (E:T) ratios of 1:1 and 1:3. Because more than 50% of NK cells express KIRs, we used as targets primary cells expressing MHC-I molecules that can inhibit KIR-expressing NK cells. Analysis of raw data did not show changes in any of these parameters (Supplementary Figure 10). In fact, there was large heterogeneity between donors. Hence, we used a similar approach as in Figure 7, measuring the average cytotoxicity in control cells for each donor and producing respective normalized values. We used these to calculate normalized values for PO-treated cells. We did not find any significant changes in cytotoxicity (Figure 8). When we analyzed degranulation, we observed a trend toward increased response at PO 10 μM at both E:T ratios. In conclusion, PO did not affect cytotoxicity, although it could facilitate NK degranulation.

## Discussion

Stimulation of the antitumor activity of the immune system is becoming a major clinical approach. Recent advances in oncology suggest that the use of clinical molecules that stimulate the immune system against infectious diseases of various origins is an alternative to traditional chemotherapy (1, 4). Foreign natural polyelectrolytes (proteins, polysaccharides, nucleic acids) and their structural analogs (polypeptides, polynucleotides) have antigenic properties and may serve as immune stimulants (5). Synthetic polyelectrolytes (SPEs) present the advantage of not being immunogenic, and the N-oxide groups of PO decrease the inherent toxicity of polyamines. This has been probed in multiple clinical settings (5, 6). Hence, PO is a clear candidate for being tested in cancer therapy. This was our first goal, and the results are encouraging, with 6 out of 20 patients responding to treatment, mainly by recruiting CD4^+^ cells to the tumor site. However, it is important to understand the mechanism of action of new drugs, and the effect of PO on lymphocytes is basically unknown. We performed an exploratory study to try to identify the molecular basis of its clinical effect.

Although PO preferentially binds to myeloid cells, it also binds to lymphocytes, although with lower affinity (7). Therefore, we studied the effects of PO *in vitro* in three immune cell subsets involved in antitumor immune responses (10). Because PO pharmacology was mainly unknown, we used multiple concentrations. Moreover, because we did not know in which functions PO could be involved, we investigated its activity in several contexts, i.e., activation, maturation, and proliferation, for each cell type analyzed, i.e., NK, T, and DC.

Although we analyzed only three HDs, we revealed that PO was immunogeneic and observed large variability: one gave a relatively strong response, one a moderate response, and the third was mainly unresponsive. This resembled our observation in breast cancer patients, with ~30% of responders. Hence, although the objective of this study was not to reveal the percentage of patients that respond to PO treatment, we believe that our results show that the effect of PO is patient/donor-dependent. This relatively low percentage of responding patients is found in most immunotherapies (28) and does not preclude its use, mainly in view of its low toxicity. New drugs usually fail in clinics due to low effect and/or high toxicity (29). The percentage of “responding” patients to a treatment is highly variable. This is more remarkable in immunotherapy because the target cell can modulate or be modulated by others or by the environment (28). For example, antibodies blocking PD-1/PD-L1 interaction are considered one of the biggest advances in cancer treatment in the last 20 years. However, this therapy improves the prognosis in only the 50% of patients with the best responder tumors. For the low responding tumors, the percentage decreases below 5%, even if patients express PD-L1 (28). Regardless of the mechanism of action and the direct effect of PO on lymphocytes, it is remarkable that this drug is barely toxic for these cells at concentrations up to 100 μg/ml, or even 500 μg/ml for T cells. This was observed even if lymphocytes were treated for several weeks. Moreover, it does not affect lymphocyte activation *in vitro*. Hence, our results *in vitro* and *in vivo* suggest that PO is a safe product.

Our results suggest that PO does not have “big” effects in the cell types and mechanisms we have investigated. This is usually seen when adjuvants are used alone (30), and although the effects of PO are small, they are coherent. For example, we observed efficient activation of several DC activation markers at certain PO concentrations (Figure 5), which correlated with the best activation of allogeneic T cells (Figure 6). Hence, DCs are clearly activated by the best immunogenic PO concentrations. Second, we observed that PO significantly increased T-cell expansion *in vitro* at the two higher concentrations, whereas the expansion of another lymphocyte lineage, i.e., NK, was not affected or decreased. Remarkably, this correlated with CD4^+^ T-cell recruitment to the tumor site in breast cancer patients.

The effects of PO were not dose-dependent. This forced us to utilize multiple concentrations under multiple conditions to unveil those conditions showing immunogenicity. In fact, the lack of a dose-dependent effect is not unusual in the immune system, where excessively strong immune activation can lead to cell inhibition. In lymphocytes, biphasic responses rely on the phosphatase CD45, which dephosphorylates the inhibitory residues of Src-kinases and leads to lymphocyte expansion and activation. However, strong CD45 activation leads to dephosphorylation of the Src-kinases activating residues, which inhibits T-cell activation (31). Hence, excessively strong activating signals can effectively lead to impaired lymphocyte activation. In addition, naïve and activated lymphocytes express different CD45 isoforms, which have different activities (22, 32). Hence, activated and naïve lymphocytes do not respond similarly to the same stimuli.

Hence, the PO cell targets that we have unveiled here could explain the variable response to this polyamine. Thus, although PO has shown its clinical value in several situations, future work should clearly establish which cancer patients can benefit from PO treatment to improve its clinical use.

This is the first study demonstrating both clinical and biological activity of PO in the domain of immune therapy for cancers. In this context, it is very interesting that two patients who responded better to PO, a complete pathological response and a partial response, suffered from triple-negative breast cancer. This type has a poor prognosis (33), and the use of PO could improve it.

## Data Availability Statement

All datasets generated for this study are included in the article/Supplementary Material.

## Ethics Statement

The studies involving human participants were reviewed and approved by N.N. Blokhin Russian Cancer Research Center in Moscow. The patients/participants provided their written informed consent to participate in this study.

## Author Contributions

CA, MC, PL-P, D-NV, YE, ED-L, and Z-YL perform the *in vitro* experiments. PL-P, JH, J-FR, and MVi design the *in vitro* experiments and wrote the manuscript. FS, OC, MVa, IV, YV, and NT perform the clinical part including collection of samples and analysis of *ex vivo* samples from Breast cancer patients.

### Conflict of Interest

The authors declare that the research was conducted in the absence of any commercial or financial relationships that could be construed as a potential conflict of interest.
