# Supplementary material for: Polyoxidonium® Activates Cytotoxic Lymphocyte Responses Through Dendritic Cell Maturation: Clinical Effects in Breast Cancer
Source: Front Immunol. 2019 Nov 28;10:2693. doi: 10.3389/fimmu.2019.02693 (PMC6892947; doi:10.3389/fimmu.2019.02693)
Supplement: Supplementary file 1 [file Data_Sheet_1.pdf]

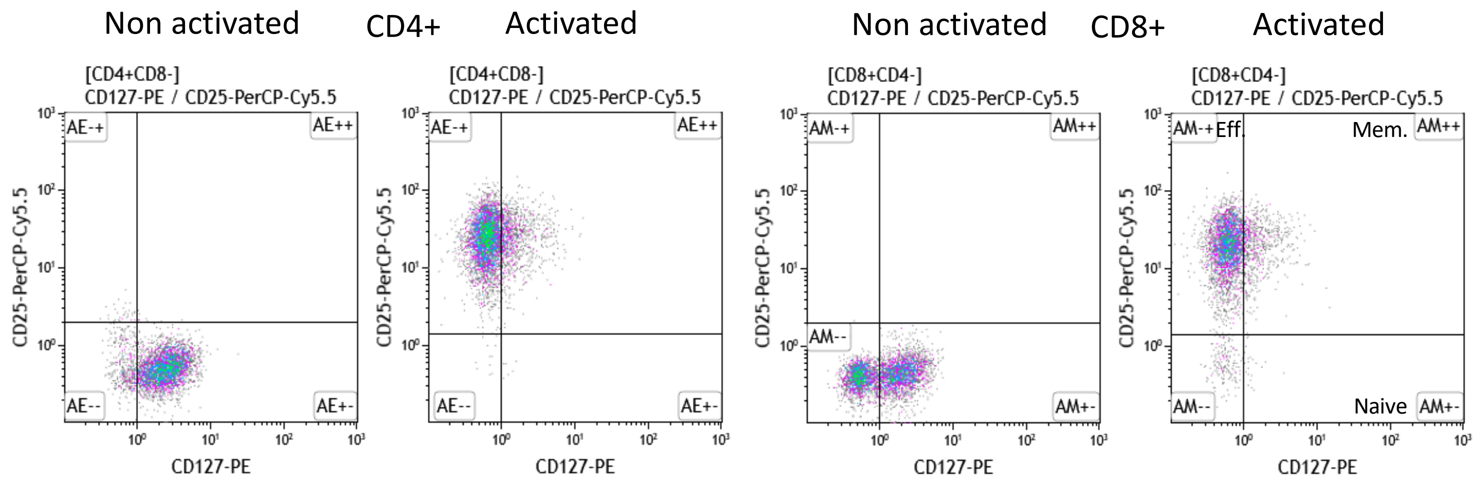

Gate	Number	%Gated
All	3 723	100,00
AE--	391	10,50
AE-+	36	0,97
AE+-	3 287	88,29
AE++	9	0,24

Gate	Number	%Gated
All	4 837	100,00
AE--	11	0,23
AE-+	3 730	77,11
AE+-	7	0,14
AE++	1 089	22,51

Gate	Number	%Gated
All	3 965	100,00
AM--	1 747	44,06
AM-+	1	0,03
AM+-	2 216	55,89
AM++	1	0,03

Gate	Number	%Gated
All	3 755	100,00
AM--	162	4,31
AM-+	2 860	76,17
AM+-	23	0,61
AM++	710	18,91

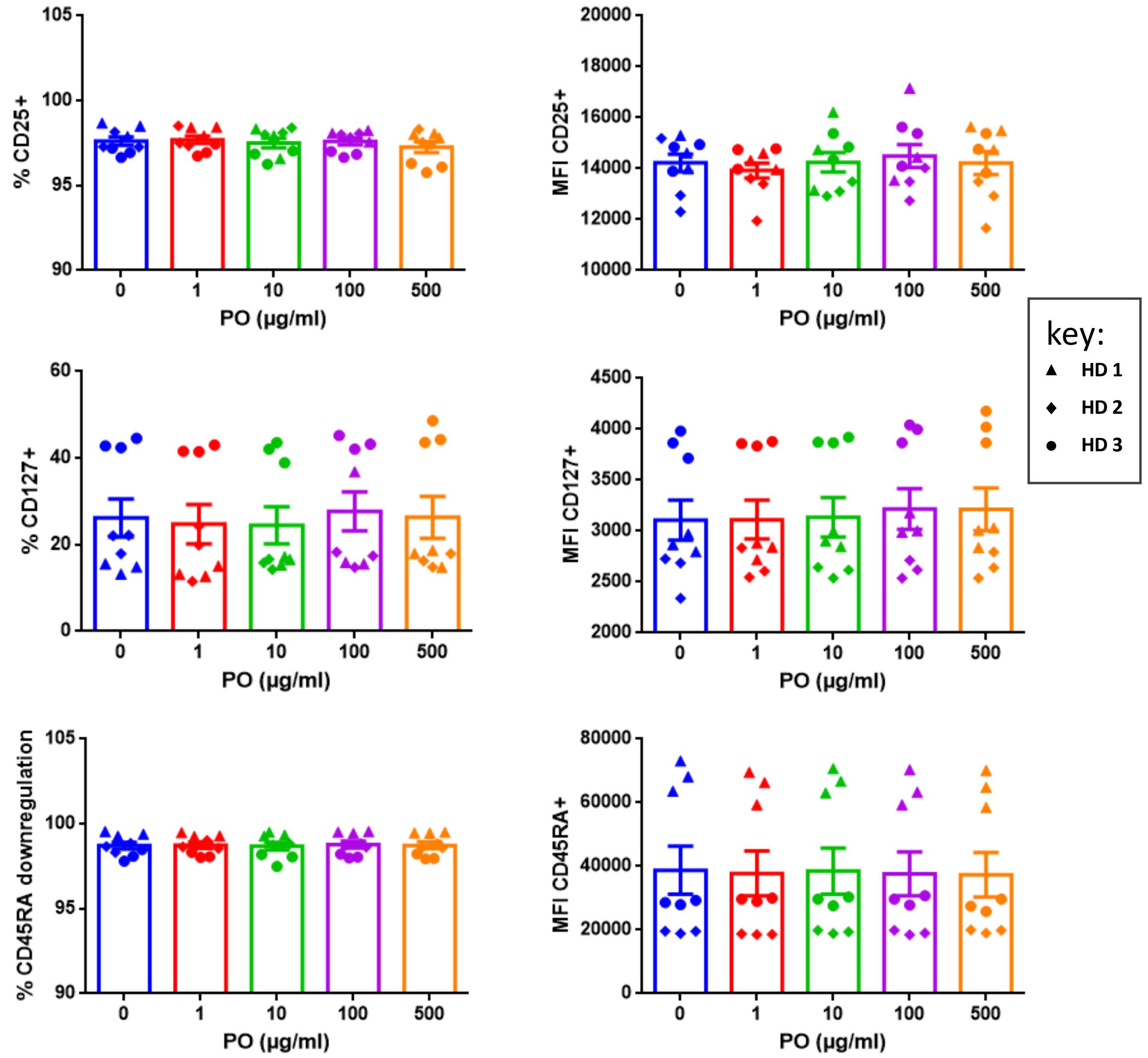

Supplemental Figure 1. A) Efficient T cell activation after CD3/CD28 costimulation. Purified T cells were activated by anti-CD3/CD28 costimulation for 5 days. CD127 and and CD25 expression were analyzed by FACs. **B) PO does not affect activation of CD4+ cells**. T cells were activated as in Supplemental Fig. 1 and the % CD25+, CD127+ and CD45RAdim cells (left) and the MFI of the positive population or CD45RAdim (right) were analyzed by FACs in the CD4+ compartment.

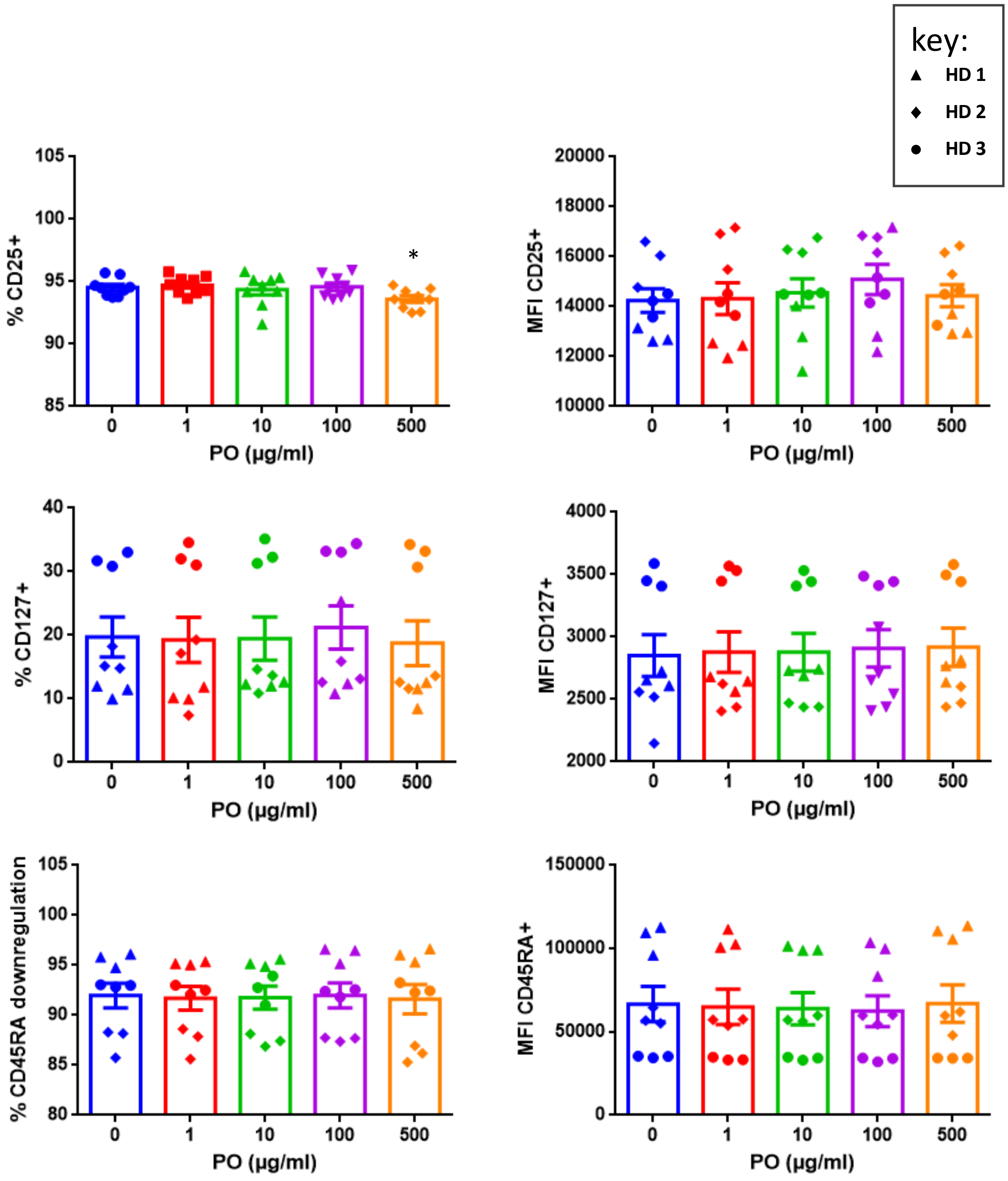

Supplemental Figure 2. PO does not affect activation of CD8+ cells. T cells were activated as in Supplemental Fig. 1 and the % CD25+, CD127+ and CD45RAdim cells (left) and the MFI of the positive population or negative (CD45RA) (right) were analyzed by FAC in the CD8+ compartment.

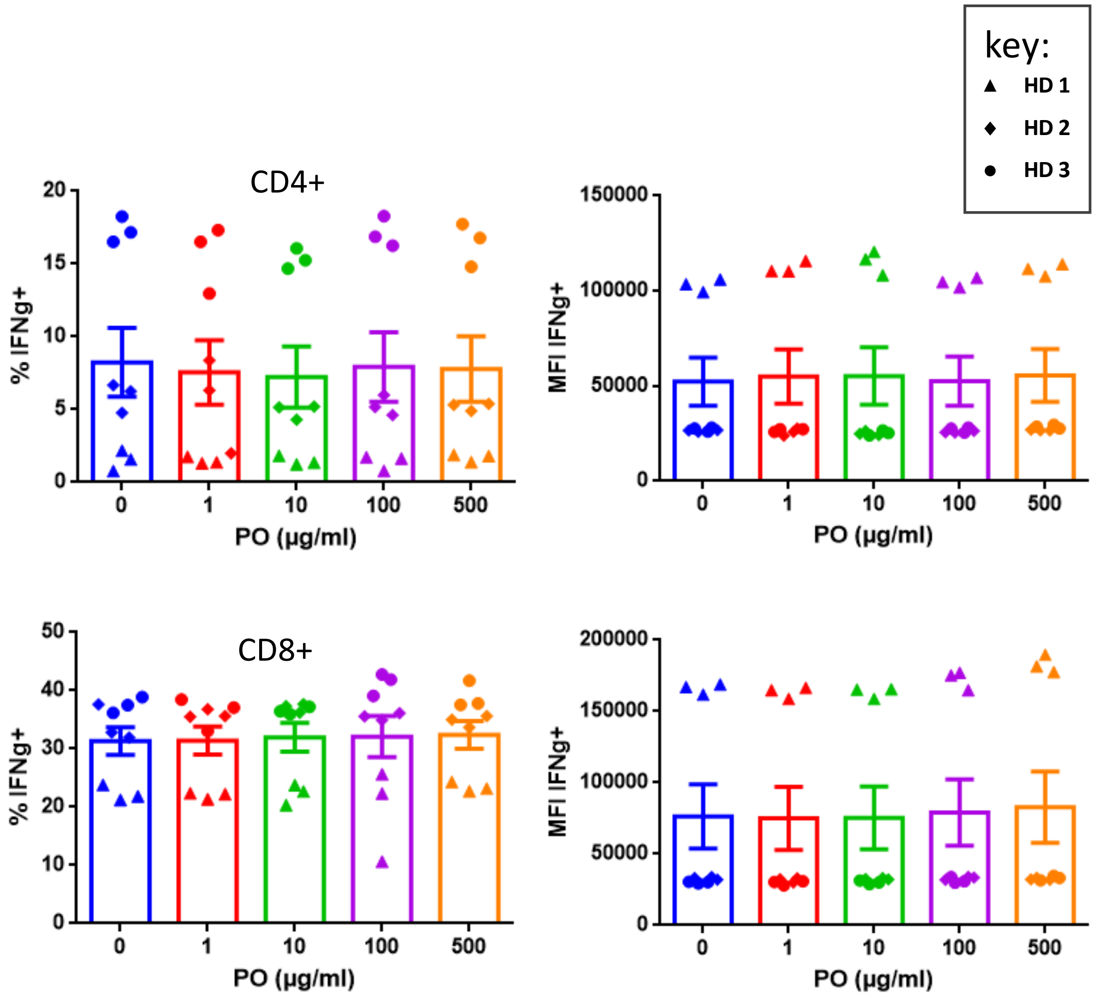

Supplemental Figure 3. PO does not affect interferon-γ expression. T cells were activated as in Fig. 1 for 5 days and then treated for 4 h with PMA (50ng/ml)/Ionomycin (1µg/ml). The % of IFNγ+ cells (left) and the MFI of the positive population were analyzed by FAC in the CD4+ (up) or CD8+ (down) compartments.

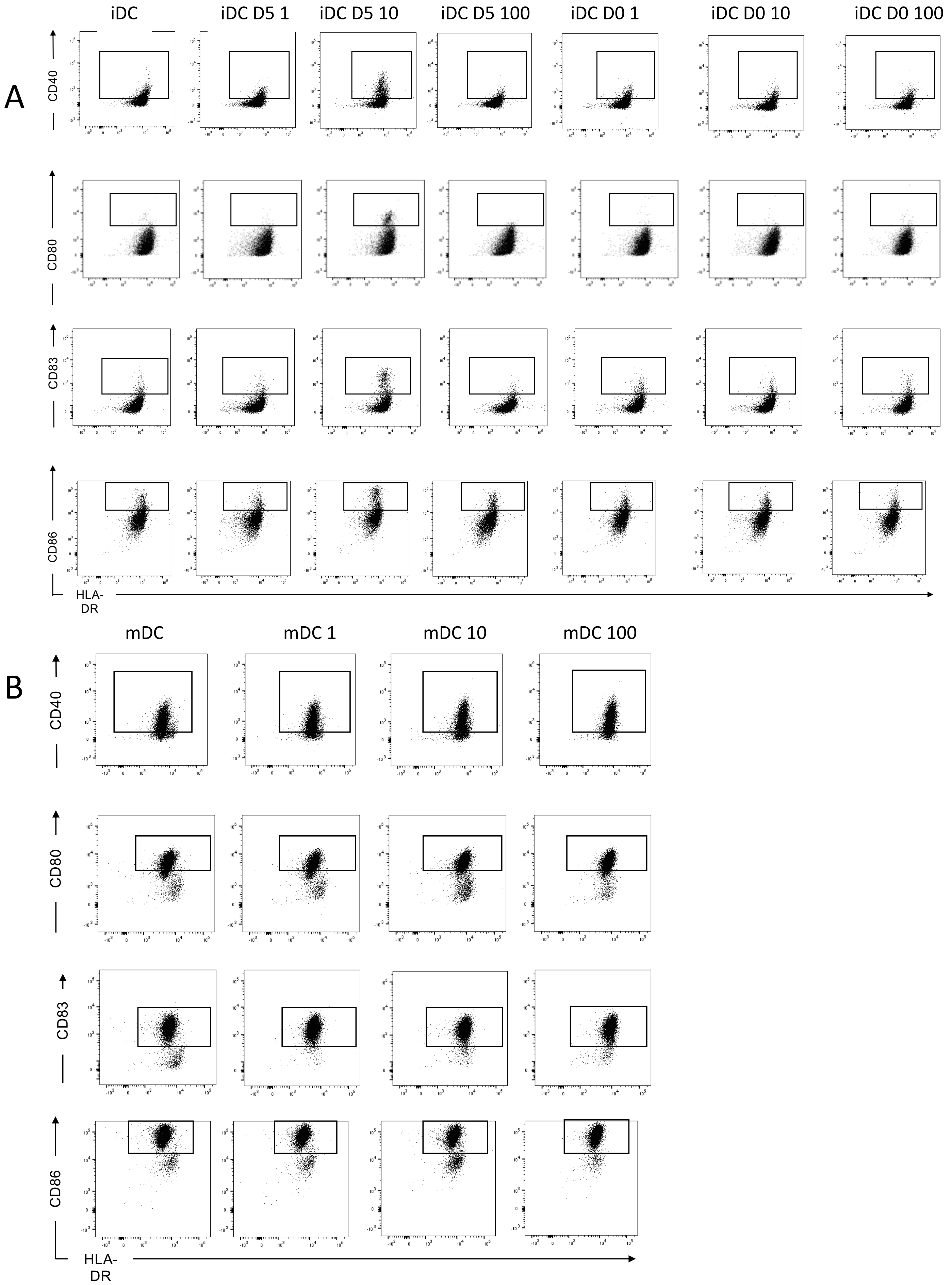

Supplemental Figure 4: Effect of PO in the expression of DC maturation markers. A) Phenotypic analyses of immature DC (iDC) generated in absence (iDC) or presence of various concentrations of PO (1, 10, 100 µg/ml). PO was added either at Day 0 (iDC D0) or Day 5 (iDC D5). **B)** Phenotypic analyses of LPS-mature DC generated in absence (mDC) or presence of various concentrations of PO from D0. Representative dot plots from 1 biological sample of donor 2 out of the 3 performed, showing CD40, CD80, CD83, CD86 (Y axes) and HLA-class II expression (X-axes).

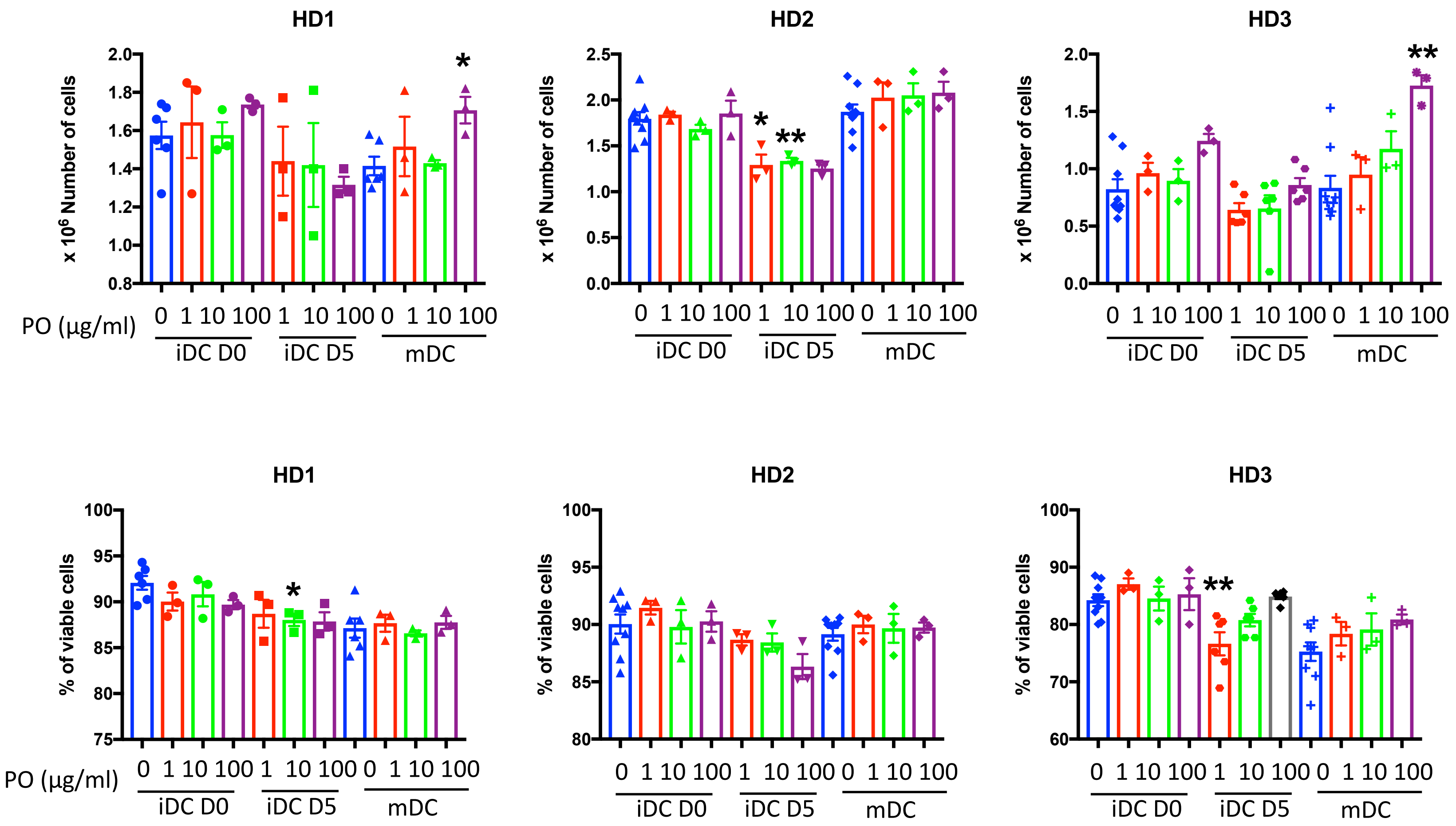

Supplemental Figure 5: PO effects on DC viability and expansion. Number of cells (top) and their viability (bottom) obtained at Day 7 from three healthy donors (HD1, HD2, HD3). Various concentrations of PO (0, 1, 10, 100 µg/ml) were added on iDC at Day 0 (iDC D0) or Day 5 (iDC D5), or on mDC. DC maturation was induced at D5 by addition of LPS (50 ng/ml) in absence or presence of various PO concentration.

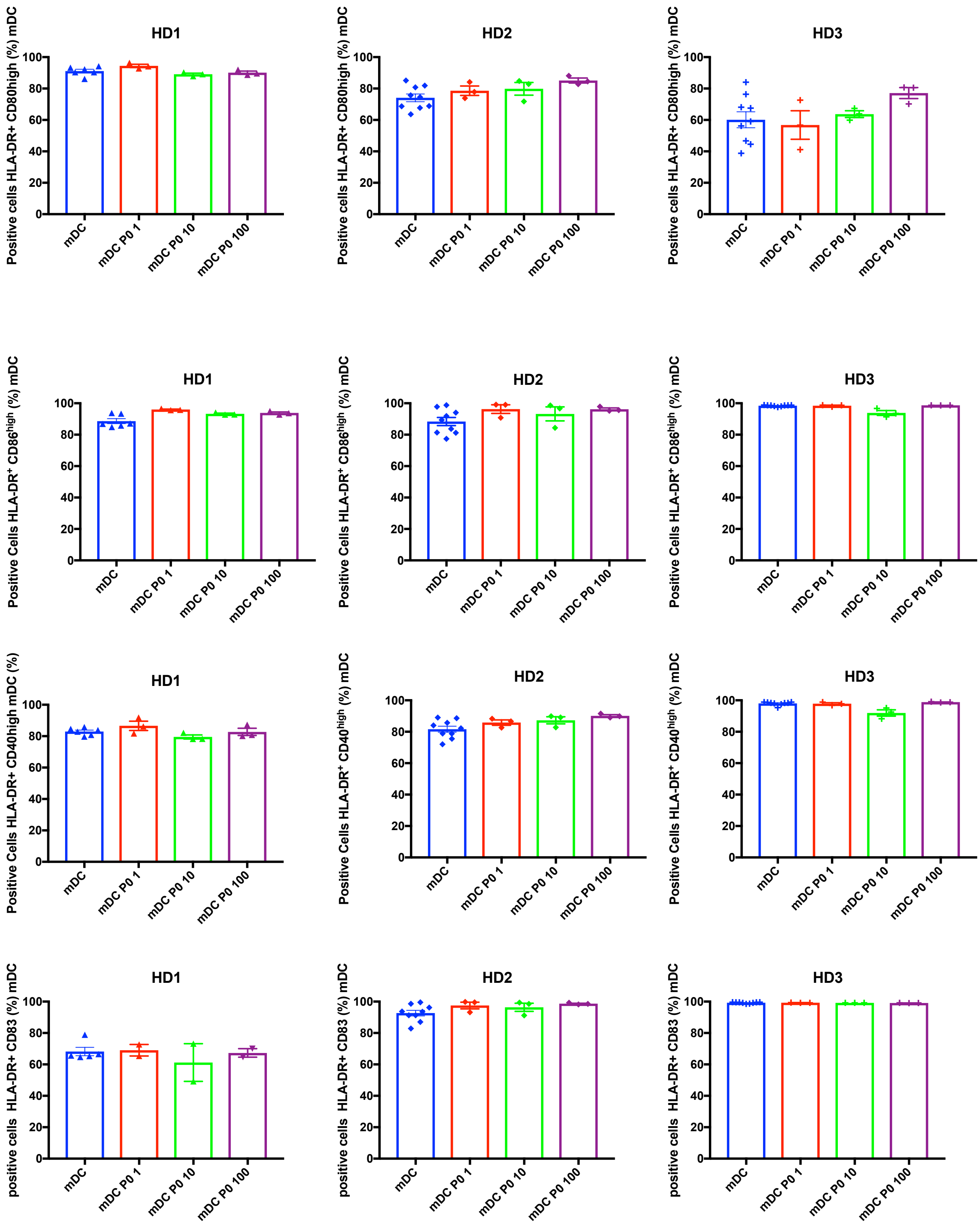

Supplemental Figure 6A: Expression of different DC maturation markers in mDC after PO treatment. mDC were induced as described in Fig. 2 and the percentage of mDC expressing the different markers was analyzed. Graphs represent means ± SEM of each independent healthy donor with experiments performed in biological triplicates.

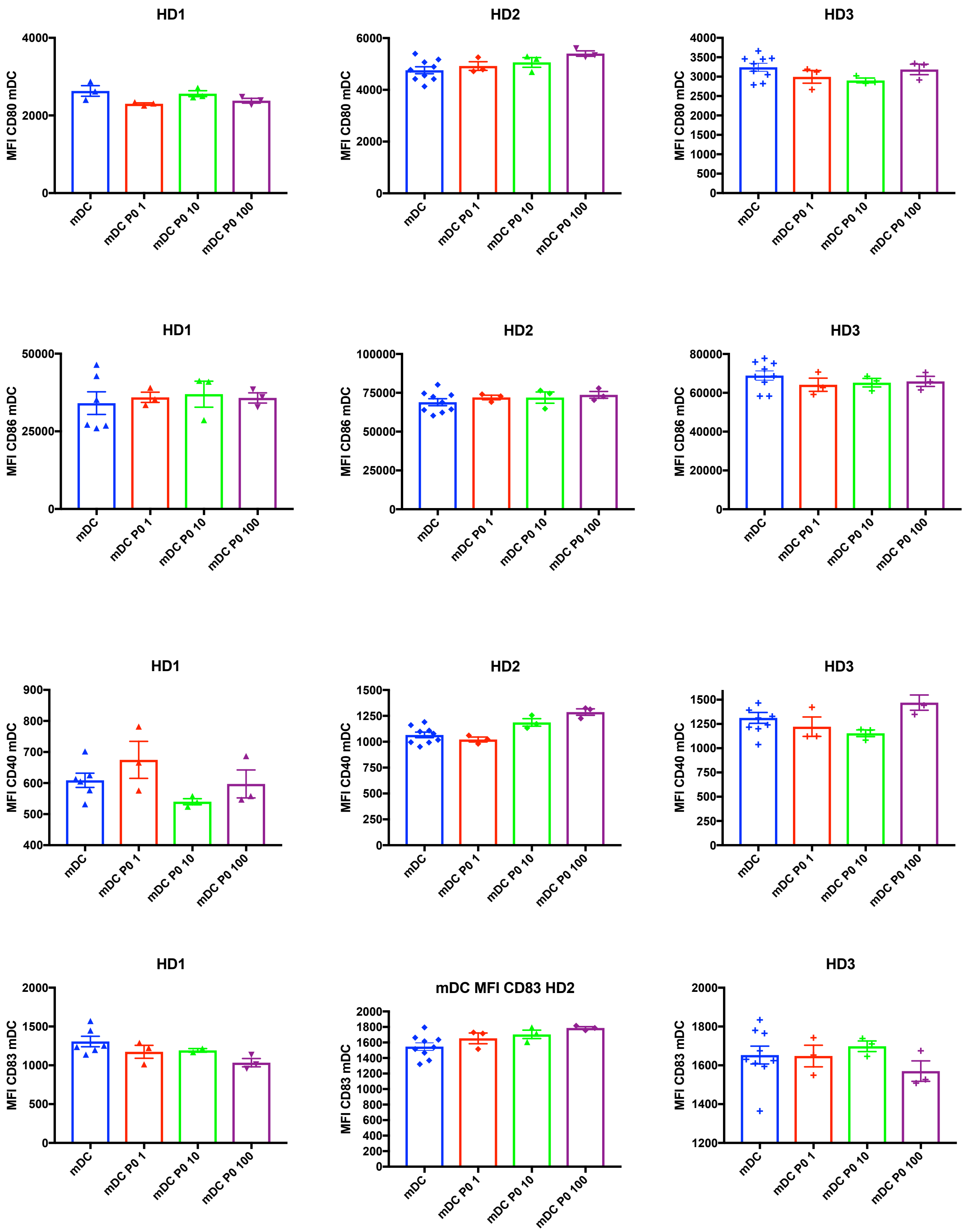

Supplemental Figure 6B: Expression of different DC maturation markers in mDC after PO treatment. mDC were induced as described in Fig. 2 and the expression level of the different markers (MFI) on mDC was analyzed. Graphs represent means ± SEM of each independent healthy donor with experiments performed in biological triplicates.

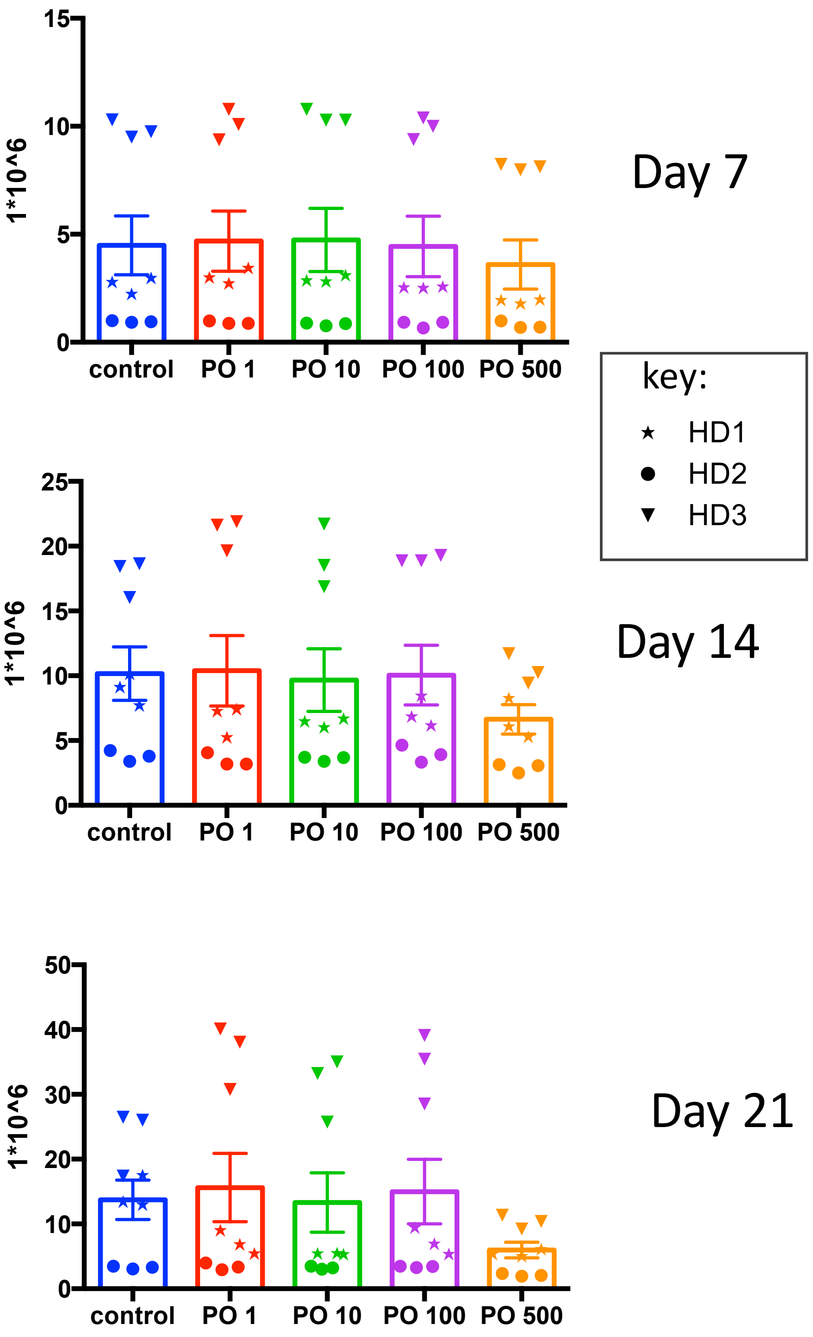

Supplemental Figure 7: High PO concentration decreases NK cell proliferation. NK cells from 3 independent healthy donors were activated by costimulation with target cells and low doses of cytokines for different times. Various concentrations of PO were added at Day 0. The number of cells were quantified at different days. Graphs represent means ± SEM; * $p<0.05$, ** $p<0.01$, *** $p<0.001$ ANOVA test compare to non-treated cells.

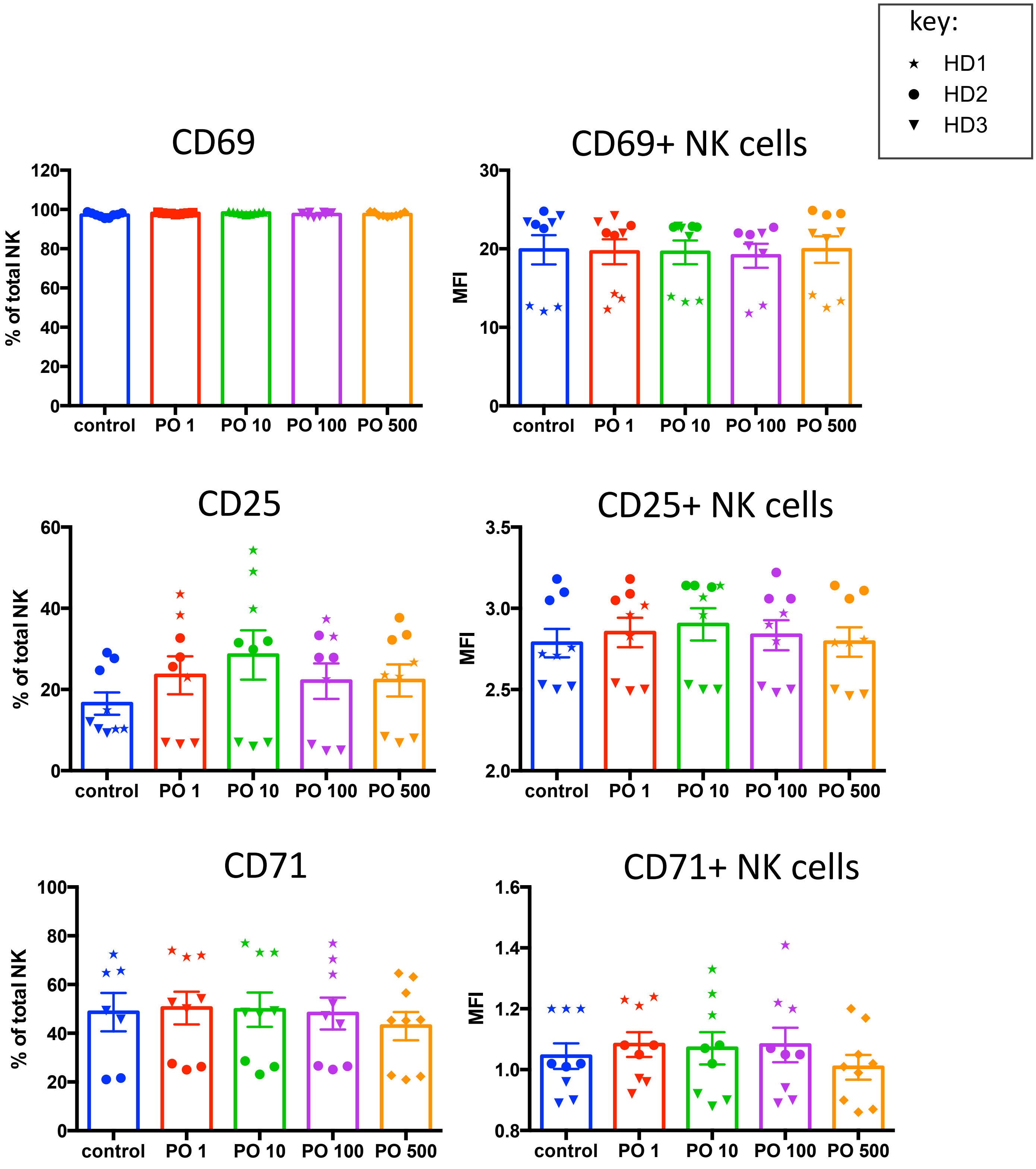

Supplemental Figure 8A: PO does not affect NK cell activation. NK cells from 3 independent healthy donors were activated by costimulation with target cells and low doses of cytokines for 21 days. Various concentrations of PO were added at Day 0. The % of positive cells for each marker or the MFI for the positive population were quantified. Graphs represent means ± SEM.

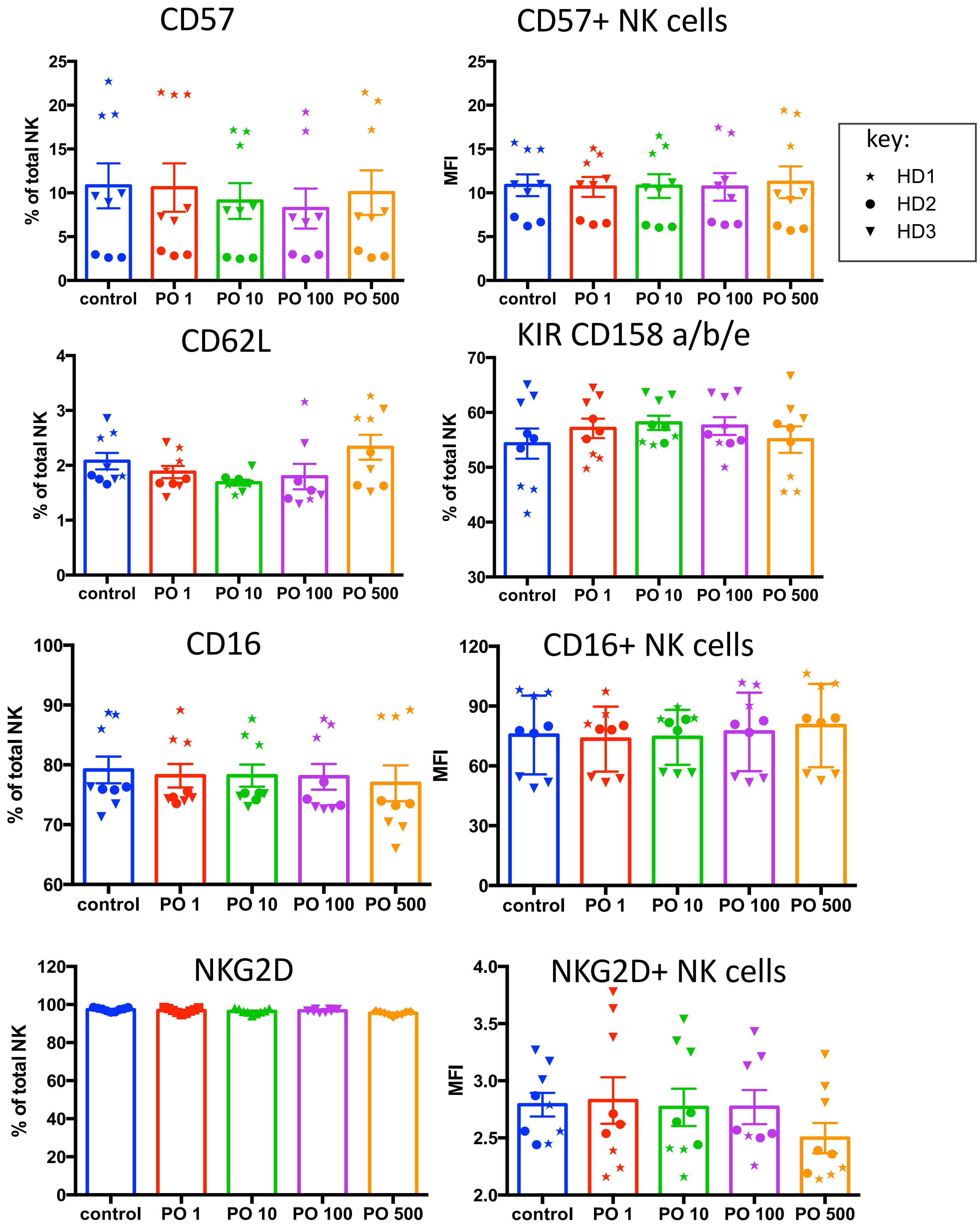

Supplemental Figure 8B: PO does not affect NK cell development. NK cells from 3 independent healthy donors were activated by costimulation with target cells and low doses of cytokines for 21 days. Various concentrations of PO were added at Day 0. The % of positive cells for each marker or the MFI for the positive population were quantified. Graphs represent means ± SEM.

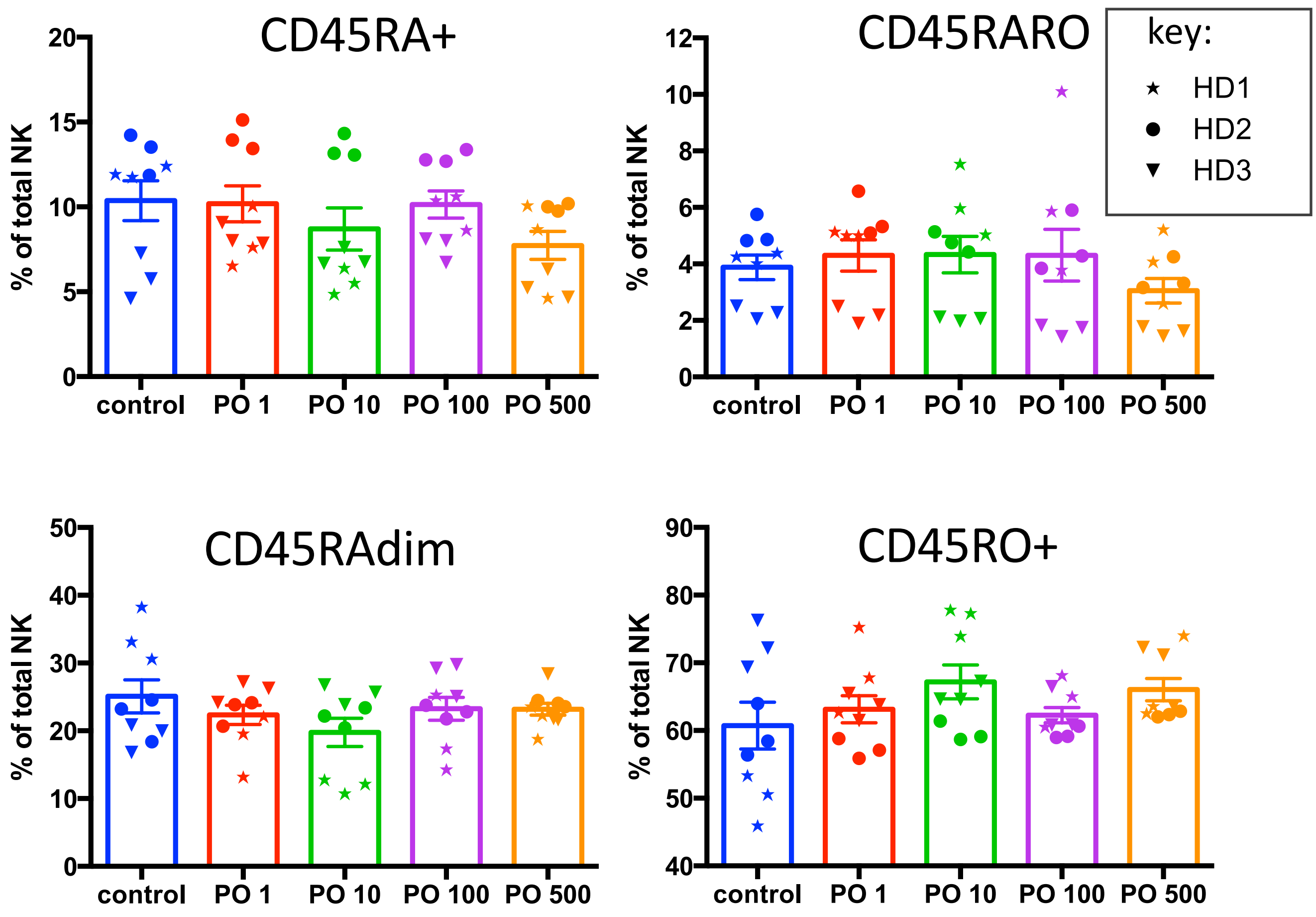

Supplemental Fig. 9: PO does not affect NK cell functional markers. NK cells from 3 independent healthy donors were activated by costimulation with target cells and low doses of cytokines for 21 days. Various concentrations of PO were added at Day 0. The % of positive cells for each marker was quantified. Graphs represent means ± SEM.

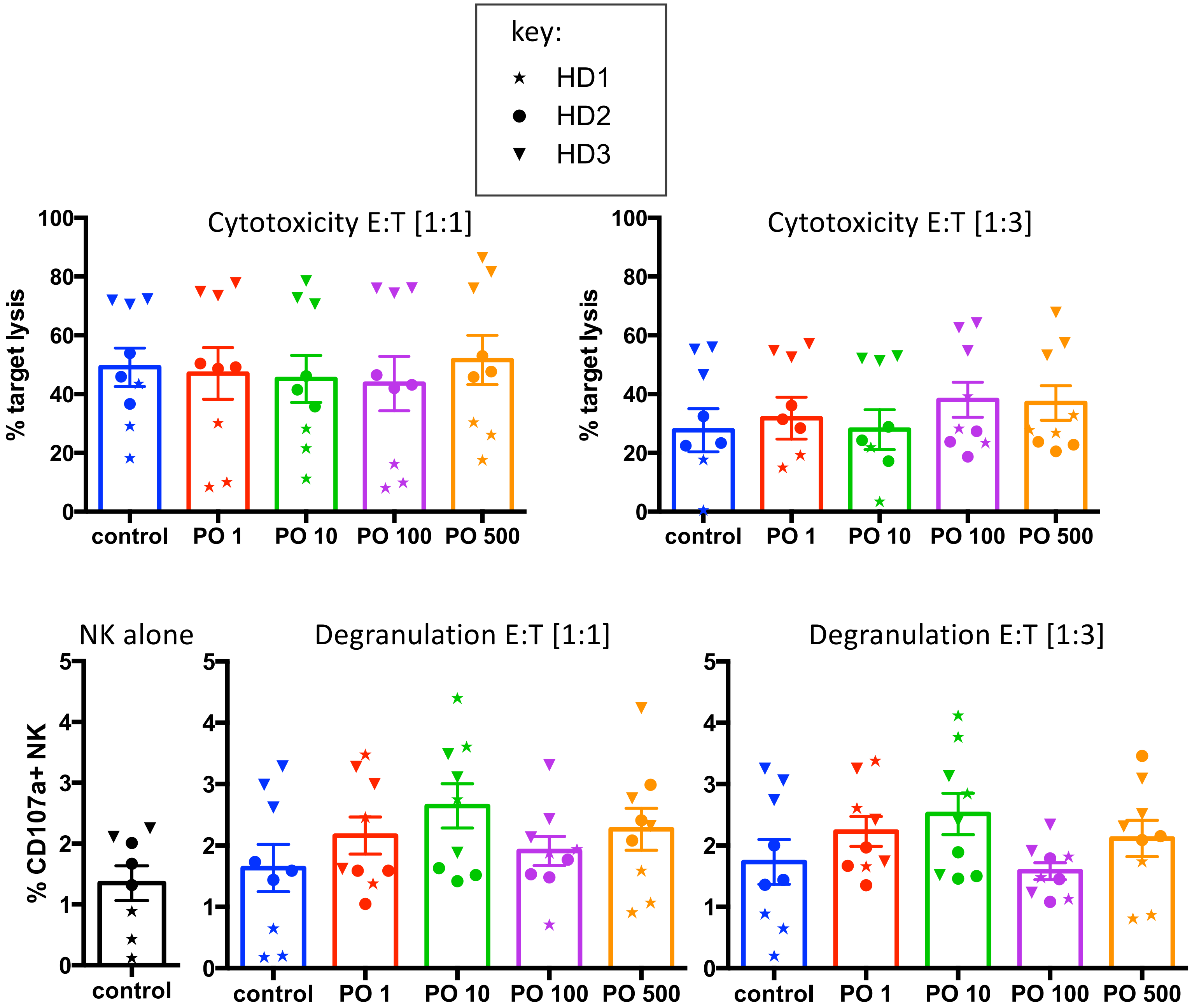

Supplemental Figure 10: PO does not affect NK cell cytolytic function. NK cells from 3 independent healthy donors were activated by costimulation with target cells and low doses of cytokines for 21 days. Various concentrations of PO were added at Day 0. Upper graphs represent toxicity against tumor cells from a B cell lymphoma patient at 2 different effector:target (E:T) ratios. Lower graphs represent the % of CD107+ cells. Graphs represent means ± SEM; * $p<0.05$, ** $p<0.01$, *** $p<0.001$ ANOVA test compare to non-treated cells.
